# Structures of Substrate Complexes of Foamy Viral Protease-Reverse Transcriptase

**DOI:** 10.1128/JVI.00848-21

**Published:** 2021-08-25

**Authors:** Marzena Nowacka, Elżbieta Nowak, Mariusz Czarnocki-Cieciura, Justyna Jackiewicz, Krzysztof Skowronek, Roman H. Szczepanowski, Birgitta M. Wöhrl, Marcin Nowotny

**Affiliations:** a Laboratory of Protein Structure, International Institute of Molecular and Cell Biology, Warsaw, Poland; b Core Facility, International Institute of Molecular and Cell Biology, Warsaw, Poland; c Lehrstuhl Biochemie IV-Biopolymere, Universität Bayreuth, Bayreuth, Germany; Ulm University Medical Center

**Keywords:** X-ray crystallography, cryo-EM, foamy viruses, protein nucleic acid complexes, reverse transcriptase

## Abstract

Reverse transcriptases (RTs) use their DNA polymerase and RNase H activities to catalyze the conversion of single-stranded RNA to double-stranded DNA (dsDNA), a crucial process for the replication of retroviruses. Foamy viruses (FVs) possess a unique RT, which is a fusion with the protease (PR) domain. The mechanism of substrate binding by this enzyme has been unknown. Here, we report a crystal structure of monomeric full-length marmoset FV (MFV) PR-RT in complex with an RNA/DNA hybrid substrate. We also describe a structure of MFV PR-RT with an RNase H deletion in complex with a dsDNA substrate in which the enzyme forms an asymmetric homodimer. Cryo-electron microscopy reconstruction of the full-length MFV PR-RT–dsDNA complex confirmed the dimeric architecture. These findings represent the first structural description of nucleic acid binding by a foamy viral RT and demonstrate its ability to change its oligomeric state depending on the type of bound nucleic acid.

**IMPORTANCE** Reverse transcriptases (RTs) are intriguing enzymes converting single-stranded RNA to dsDNA. Their activity is essential for retroviruses, which are divided into two subfamilies differing significantly in their life cycles: *Orthoretrovirinae* and *Spumaretrovirinae*. The latter family is much more ancient and comprises five genera. A unique feature of foamy viral RTs is that they contain N-terminal protease (PR) domains, which are not present in orthoretroviral enzymes. So far, no structural information for full-length foamy viral PR-RT interacting with nucleic substrates has been reported. Here, we present crystal and cryo-electron microscopy structures of marmoset foamy virus (MFV) PR-RT. These structures revealed the mode of binding of RNA/DNA and dsDNA substrates. Moreover, unexpectedly, the structures and biochemical data showed that foamy viral PR-RT can adopt both a monomeric configuration, which is observed in our structures in the presence of an RNA/DNA hybrid, and an asymmetric dimer arrangement, which we observed in the presence of dsDNA.

## INTRODUCTION

Reverse transcription involves the conversion of single-stranded RNA into double-stranded DNA (dsDNA). It is a key step in the proliferation of retroviruses and retrotransposons and is catalyzed by the enzyme reverse transcriptase (RT) (1). Reverse transcriptases have two activities: DNA polymerase (POL) synthesizes DNA on DNA or RNA templates, whereas RNase H (RH) degrades the RNA strand of RNA/DNA hybrid intermediates. Retroviruses are divided into two subfamilies: *Orthoretrovirinae* (OV), which include six genera, one of which is lentiviruses, such as human immunodeficiency virus type 1 (HIV-1), and *Spumaretrovirinae*, which consist of five genera (2–4). Foamy viruses (FVs) have many characteristics that set them apart from OVs, including differences in the proliferation cycle (5–7) and a different domain structure of the mature RT, which, in addition to POL and RH, harbors the N-terminal protease (PR) domain (PR-RT) (8).

HIV-1 RT is a heterodimer that consists of a larger subunit (p66) and a smaller subunit that lacks the RNase H domain (p51) (9). p66 comprises a POL domain, followed by the connection and RH domains, the latter of which is rigidly placed within the heterodimer. The POL domain resembles a right hand, with subdomains, termed fingers, that stabilize the template; a palm that comprises the POL active site; and a thumb that stabilizes the substrate. The p51 subunit lacks POL activity and is thought to play only a structural role.

Crystal structures have been determined for the monomeric form of RT from Moloney murine leukemia virus (MoMLV) (10, 11). For the nearly identical enzyme from xenotropic mouse Moloney leukemia-related virus (XMRV), structures are available for a POL-connection fragment with a bound RNA/DNA substrate (12) and an isolated RH domain (13). The RNase H domain of MoMLV/XMRV RT is tethered to the rest of the protein through a flexible linker. Thus, it is mobile and only occasionally interacts with the hybrid to execute cleavage (12). Therefore, the mechanisms of action of the XMRV and HIV-1 RH domains are different.

For long terminal repeat (LTR) retrotransposon RTs, structural information is limited to yeast Ty3 RT in complex with an RNA/DNA substrate (14). It has been shown to adopt an unexpected asymmetric homodimer architecture, with one subunit in an active POL configuration and the other resembling the conformation of the HIV-1 RT p51 subunit and contributing the RNase H activity. A key difference between retrotransposon and retroviral RTs is that the original RH domain of retrotransposon RT in viral enzymes is converted to the inactive connection domain, whereas a new RH domain of cellular origin is added to the protein (15).

For FVs, nuclear magnetic resonance (NMR) structures for the isolated prototype FV (PFV) RH domain (16, 17) and the simian FV from macaque (SFVmcy [formerly SFVmac]) PR domain have been solved (18). The isolated PR domain is monomeric in solution but forms transient dimers (19). During virus assembly, the formation of catalytically active PR dimers occurs via the binding of PR-RT to a specific region on the FV pregenomic RNA, called the PR-activating RNA motif (PARM) (20) (reviewed in reference 8). Retroviral PRs are members of the well-characterized family of aspartic PRs and have been shown to be active as dimers (21). To create the active site, each subunit of the homodimer provides one catalytic aspartate residue situated in the conserved motif Asp-Thr/Ser-Gly (22, 23). In addition, other regions of the PR, i.e., the C and N termini and the so-called flap region on top of the active site, contribute to dimer formation (21). To date, no structure of full-length FV PR-RT in complex with a nucleic acid substrate has been reported. Here, we present crystal and cryo-electron microscopy (cryo-EM) structures of FV PR-RT that were solved using the protein from marmoset FV (MFV). The structures revealed an unexpected ability of FV PR-RT to adopt monomeric and dimeric configurations, depending on the type of bound substrate.

## RESULTS AND DISCUSSION

### Crystal structures reveal two types of MFV PR-RT complexes with nucleic acids.

Our first aim was to solve a crystal structure of an FV PR-RT. We sought to reveal the molecular architecture of an RT that comprises a PR domain and elucidate its mechanism of nucleic acid substrate binding. We generated expression constructs for seven PR-RTs from various FVs. Multiple constructs were prepared that comprised full-length protein with PR, POL, and RH domains and truncated variants that lacked either the PR or RH domain. Proteins for nine different constructs were efficiently expressed in bacteria in a soluble form. They were purified and underwent extensive crystallization trials in the presence of double-stranded DNAs (dsDNAs) or RNA/DNA hybrids of various lengths and sequences. All of these substrates contained 5′ overhangs in one DNA strand of dsDNA or an RNA strand in RNA/DNA hybrids to direct the recessed 3′ terminus of the nucleic acid to the POL active site. Some hybrids comprised the polypurine tract (PPT) sequence of the respective FVs. Polypurine tracts are special sequences that are left intact by RNase H in a key step of reverse transcription such that they can serve as primers for the synthesis of the second strand of DNA, called plus-strand DNA [(+)DNA] (24). PPT sequences have been successfully used for the crystallization of XMRV (12) and Ty3 (14) RTs.

**FIG 1:**
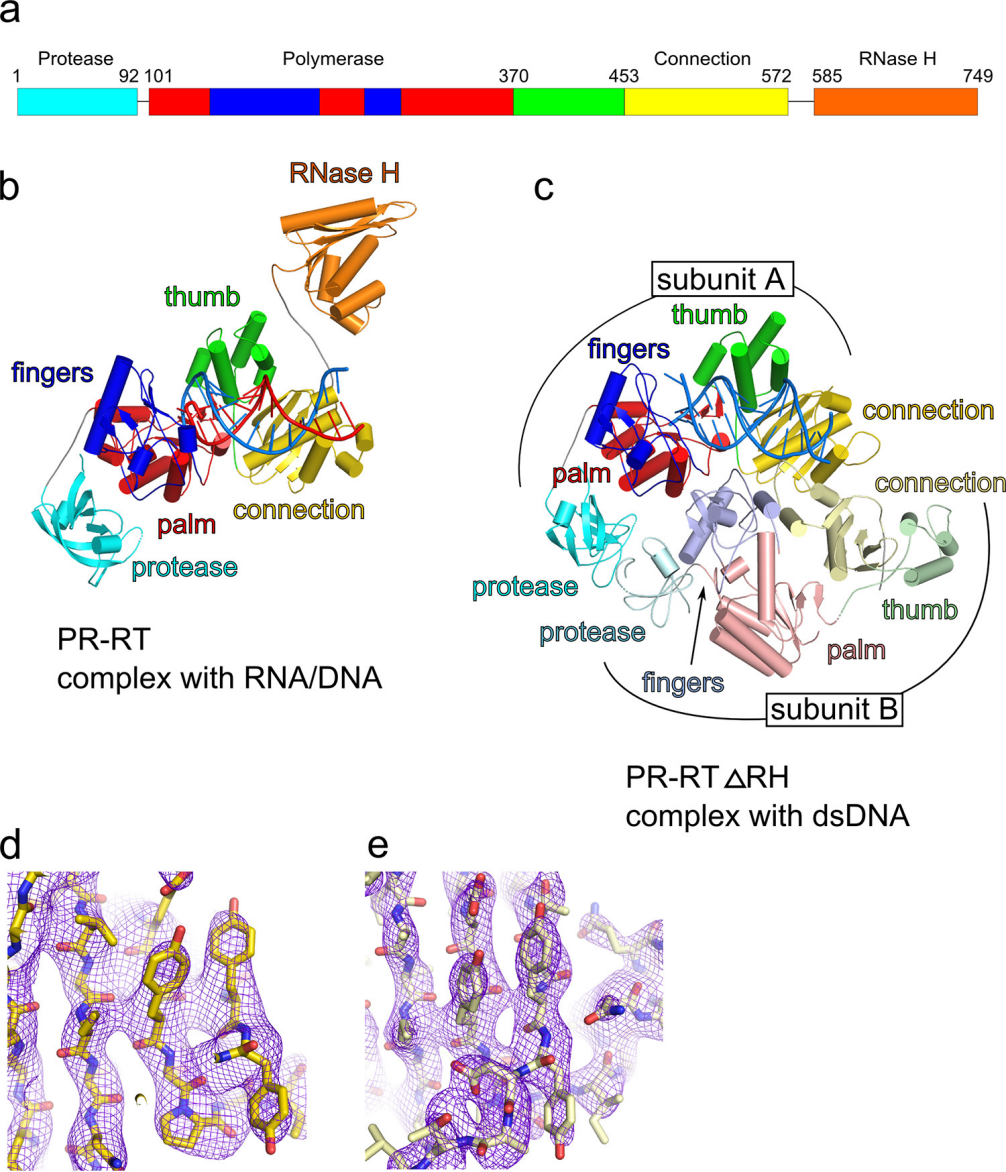
Crystal structures of nucleic acid complexes of MFV PR-RT. (a) Schematic diagram of the domain composition of MFV PR-RT with the residue numbers at the (sub)domain boundaries. The schematic diagram is shown in cyan for the protease domain, blue for the finger subdomain of the polymerase, red for the palm subdomain, green for the thumb subdomain, yellow for the connection domain, and orange for the RNase H domain. (b) Cartoon representation of MFV PR-RT in complex with an RNA/DNA hybrid substrate. (c) Structure of MFV PR-RT ΔRH in complex with dsDNA. Lighter shades of colors are used for domains/subdomains of subunit B. (d and e) Fragment of the connection domain. (d) Structure of MFV PR-RT in complex with RNA/DNA. (e) Structure of MFV PR-RT ΔRH in complex with dsDNA (protein subunit B). A 2*F*_o_-*F*_c_ simulated annealing composite omit electron density map contoured at 1.0 σ (d) and 1.2 σ (e) is shown as purple mesh.

From these crystallization trials, we obtained numerous hits, many of which were optimized to obtain crystals of the size that is required for diffraction experiments. However, only two types of crystals diffracted X-rays to resolutions that were sufficient for structure solution. Both of these crystal types were obtained with MFV PR-RT. The first comprised the full-length PR-RT protein (Fig. 1a) and an RNA/DNA hybrid with 15 bp and overhangs in the RNA strand (a 2-nucleotide [nt] overhang at the 5′ terminus and a 1-nt overhang at the 3′ end [substrate 1]). The sequence of this substrate corresponded to the PPT sequence with the preferred RNase H cut site that was located 13 nt from the recessed 3′ end of the primer (see Materials and Methods). These crystals belonged to the *P* 4_3_2_1_2 space group. Their variant, which was grown using selenomethionine (SeMet)-substituted protein, diffracted X rays to a 3.25-Å resolution at a synchrotron source. The structure was solved using selenium single anomalous diffraction (SAD), and the structure was built using a homology model that was based on the XMRV RT protein structure (PDB accession no. 4HKQ) (12). The structure was refined against data that were collected for native protein crystals that diffracted X rays to a 3.1-Å resolution at the synchrotron source (Fig. 1b). The second crystal form was obtained using a deletion variant of MFV PR-RT that lacked the RH domain (MFV PR-RT ΔRH) and was grown in the presence of 13-bp dsDNA with a 5′ overhang of 2 nt in one strand (substrate 2) (see Materials and Methods for the sequence). These crystals belonged to the *C* 2 space group and diffracted X-rays to a 3.1-Å resolution at the synchrotron source. The structure was solved by molecular replacement using a model of MFV PR-RT from the RNA/DNA complex structure in which the RH domain was removed (Fig. 1c). Sample electron density maps are shown in Fig. 1d and e. The data collection and refinement statistics are shown in Table 1.

**TABLE 1:** X-ray data collection and refinement statistics

Parameter	Value		
	MFV PR-RT in complex with RNA/DNA (SeMet)	MFV PR-RT in complex with RNA/DNA (native)	MFV PR-RT ΔRH in complex with dsDNA
Data collection statistics^*a*^			
Beamline	P13 EMBL	P13 EMBL	P11 DESY
Space group	*P* 4_3_2_1_2	*P* 4_3_2_1_2	*C* 2
Cell dimensions			
*a*, *b*, *c* (Å)	107.1, 107.1, 252.2	105.0, 105.0, 253.5	158.5, 107.6, 118.1
α, β, γ (°)	90.0, 90.0, 90.0	90.0, 90.0, 90.0	90.0, 98.7, 90.0
Resolution (Å)	50.0–3.25 (3.5–3.25)	50.0–3.1 (3.28–3.1)	50.0–3.09 (3.28–3.09)
*R*_merge_	0.141 (0.857)	0.074 (1.434)	0.126 (1.143)
*I*/σ*I*	15.5 (1.7)	21.1 (1.5)	9.3 (1.2)
CC_1/2_	99.8 (99.6)	99.9 (82.1)	99.6 (56.4)
Completeness (%)	91.9 (86.1)	99.8 (99.3)	99.4 (98.0)
Redundancy	13.9 (13.5)	13.1 (13.5)	3.8 (3.8)

Refinement statistics			
Resolution (Å)		48.5–3.1	48.8–3.09
No. of reflections		26,640	35,690
*R*_work_ (%)		24.74	22.86
*R*_free_ (%)		27.30	28.25
No. of atoms		6,270	8,959
Protein		5,569	8,390
Ligand/ion		700	569
Water		1	
*B* factors (Å^2^)			
Protein		126.6	109.5
Ligand/ion		128.4	84.0
Water		85.8	
Root mean square deviations			
Bond lengths (Å)		0.003	0.003
Bond angles (°)		0.59	0.72

a The data collection statistics are based on a single crystal. Values in parentheses are for the highest-resolution shell. CC_1/2_, correlation coefficient between the average intensities in two parts of the unmerged data, each with a random half of the measurements of each unique reflection (52).

In the structure of full-length PR-RT, the electron density was observed for all four domains: PR, POL, connection, and RNase H (Fig. 1a and b). The POL-connection part forms a globular core of the enzyme that is very similar to the p66 subunit of HIV-1 RT and XMRV RT. The POL domain of p66 HIV-1 RT (PDB accession no. 4PQU) (25) can be superimposed on the POL domain from MFV PR-RT with a root mean square deviation (RMSD) of 3.3 Å for the positions of 225 C_α_ atoms. The POL domain of XMRV RT (PDB accession no. 4HKQ) (12) can be superimposed with an RMSD of 2.0 Å over 247 C_α_ atoms. Additionally, the N-terminal PR domain that comprises a six-stranded β-barrel is visible in the structure (residues 4 to 93). Its structure is very similar to the previously determined solution NMR structure of the separate SFV PR (PDB accession no. 2JYS [18]) (RMSD of 1.0 Å over 74 C_α_ atoms). The unique element of MFV PR-RT that is not present in RT structures that have been solved to date is formed by a region between the POL and PR domains (residues 93 to 120). It consists of a linker that lacks a secondary structure and an α-helix. This region does not exhibit sequence homology to retroviral PRs or any other RT. However, studies with deletion variants implied that it is an intrinsic part of the RT domain that is necessary for the solubility and integrity of the protein (26).

The RH domain is linked to the connection domain with a flexible linker (residues 573 to 584). In the previously determined structure of XMRV RT, the mobile RH domain was not visible, although it was present in the crystal (12). However, in the present MFV PR-RT structure, the RH domain is well defined (Fig. 1b) because of its stabilization by crystal contacts. It adopts a typical RNase H structure with a five-stranded antiparallel central β-sheet and five α-helices with a characteristic basic protrusion element that is involved in substrate binding (17). The RH domain from our structure is very similar to the previously determined solution structure of PFV RNase H (PDB accession no. 2LSN [17]) (RMSD of 1.5 Å over 141 C_α_ atoms). It is also quite similar to the XMRV RH domain (PDB accession no. 3V1O [13]) (RMSD of 2.3 Å over 117 C_α_ atoms) and human RNase H1 (PDB accession no. 2QK9 [27]) (RMSD of 2.5 Å over 102 C_α_ atoms), all of which also possess the basic protrusion.

In the structure, we observed the electron density for 17 nt of the RNA strand and 15 nt of the DNA strand of the hybrid substrate. It is bound by the POL-connection fragment of MFV PR-RT in a manner that is similar to those of other RTs (HIV-1, XMRV, and Ty3), with the 3′ recessed end of the nucleic acid duplex located at the active site of the POL domain.

**FIG 2:**
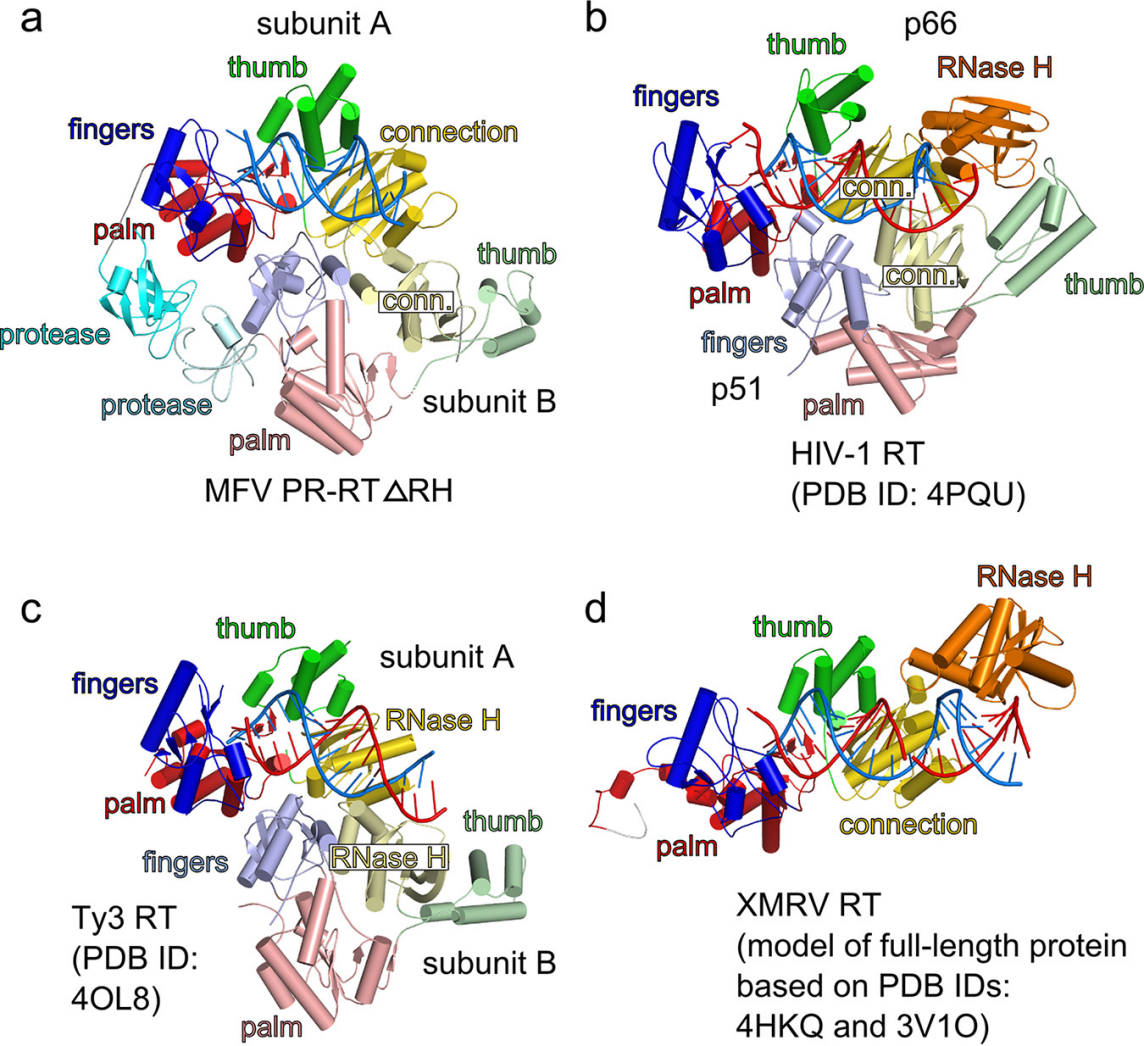
Comparison of structures of various LTR reverse transcriptases. The subdomains/domains are colored as described in the legend of Fig. 1. RNA strands are in a red ladder representation, and DNA is in a blue ladder representation. (a) MFV PR-RT ΔRH bound to dsDNA (present study). (b) HIV-1 RT in complex with RNA/DNA (PDB accession no. 4PQU) (25). (c) Ty3 RT bound to RNA/DNA (PDB accession no. 4OL8) (14). (d) Small-angle X-ray scattering-based model of full-length XMRV RT that was prepared based on the structure of the POL-connection fragment bound to an RNA/DNA hybrid substrate (PDB accession no. 4HKQ) (12) and the isolated XMRV RNase H domain structure (PDB accession no. 3V1O) (13).

**FIG 3:**
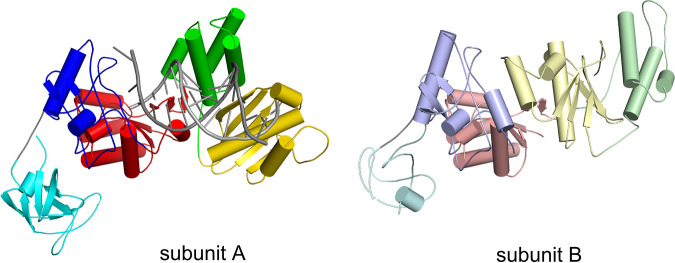
Comparison of structures of subunits A and B of MFV PR-RT ΔRH in complex with dsDNA. The structure is shown in a cartoon representation and colored as described in the legend of Fig. 1. In the left panel, the nucleic acid is shown as a gray ladder.

The other structure that is reported here is for a ΔRH variant of MFV PR-RT in complex with dsDNA. Early during model building, it became apparent that the architecture of the macromolecular assembly in this structure is different from that of the full-length MFV PR-RT structure. Biochemical characterization of the enzyme suggested that FV PR-RTs are monomeric enzymes (18, 28). Unexpectedly, the complete model of MFV PR-RT ΔRH revealed a dimer of the protein that interacted with the dsDNA duplex (Fig. 1c). Moreover, the arrangement of this dimer was very similar to those of other dimeric RTs, HIV-1 RT and Ty3 RT. For the latter, an important difference is that the MFV connection domain corresponds to the Ty3 RNase H domain (Fig. 2). One subunit that we termed subunit A adopts a structure that is analogous to the monomeric form that is observed in the full-length MFV PR-RT hybrid complex structure (RMSD of 2.6 Å over 477 C_α_ atoms), with small differences in the positions of the individual (sub)domains. However, the conformation of the other subunit (subunit B) is markedly different. The palm/finger module remains largely the same, but the position of the finger/thumb module is completely rearranged such that it blocks the POL active site (Fig. 3). This conformation is very similar to the ones that were observed for the p51 subunit of HIV-1 RT and subunit B of Ty3 RT, with the largest differences in the positioning of the thumb subdomain. The fact that this dimeric arrangement of MFV PR-RT is very similar to those of other RTs strongly implies that this architecture is a native state that is adopted by the protein and not a crystallization artifact.

**FIG 4:**
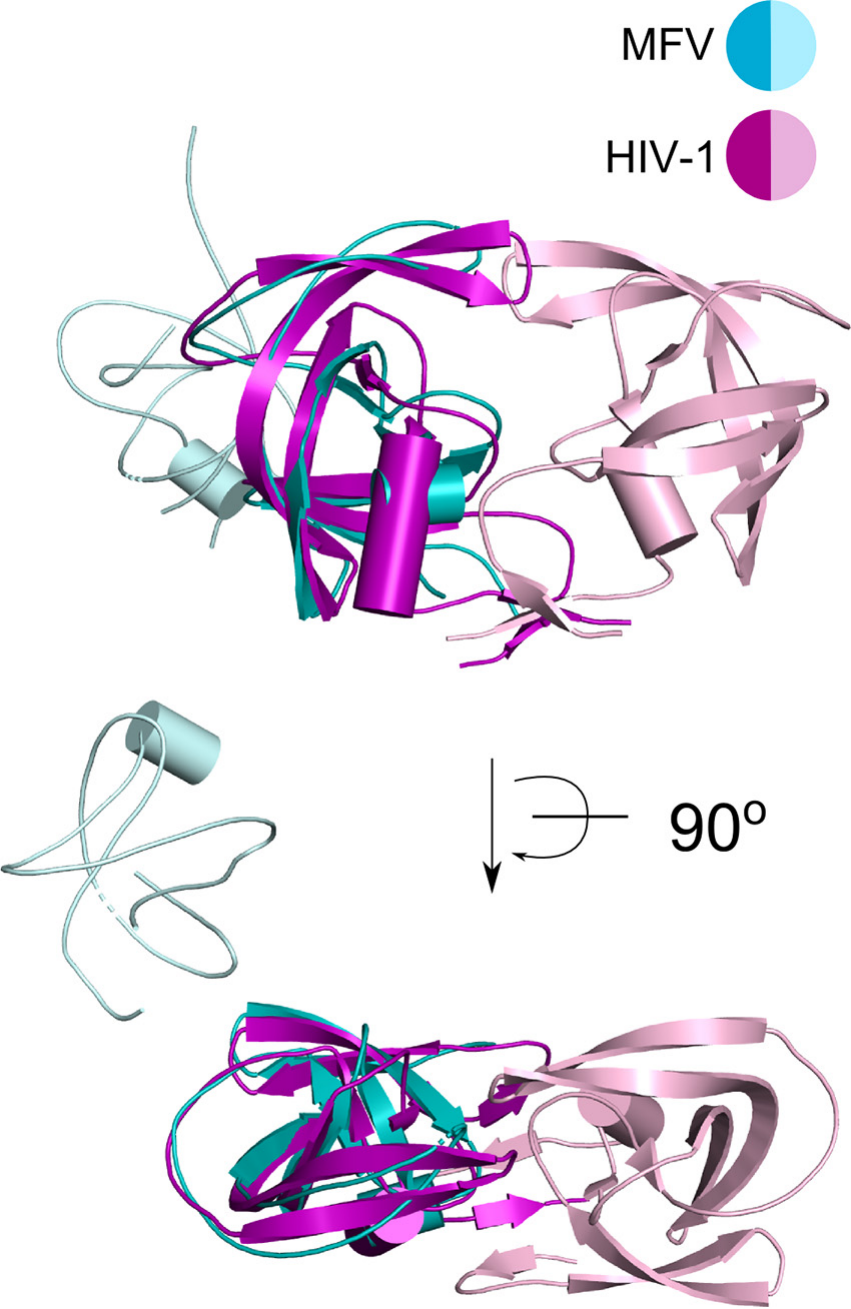
Comparison of the MFV PR domain dimer and the HIV-1 PR dimer. PR domains from the MFV PR-RT ΔRH structure are in shades of cyan. The PR domain from subunit A is superimposed on one HIV-1 PR protomer (in purple; the other protomer is in pink) (PDB accession no. 1KJF) (51). Two views that are rotated by 90° around the *x* axis are shown.

In the structure of MFV PR-RT ΔRH, electron density was observed for 13 bp of the dsDNA and a 2-nt overhang. Similar to other RT structures, the recessed 3′ end of the DNA interacts with the POL active site of subunit A. The PR domains of both subunits are also visible in the structure. Interestingly, they interact with each other. Retroviral PRs are known to function as obligate dimers (21). However, the dimeric arrangement of the PR domains in our structure is markedly different from the architecture of stand-alone PRs such as the HIV-1 enzyme and would not allow active-site formation (Fig. 4) (29). An active dimer configuration was also not observed in crystal contacts. The PR domain has a similar position relative to the rest of the protein in the monomeric RNA/DNA complex structure and in subunit A of the dimeric dsDNA complex (Fig. 1b and c). This implies that the formation of an active dimeric form of MFV PR, which would be similar to that of the HIV-1 PR dimer, would require a rearrangement of the entire RT structure to a configuration that is different from the one observed in our dsDNA complex structure. Further studies are needed to elucidate the nature of this configuration.

In conclusion, our structures revealed the architecture of PR-containing MFV RT in two configurations: an RNA/DNA-bound protein monomer form and a dsDNA-bound asymmetric homodimer.

### The structures reveal the mechanism of substrate binding by MFV PR-RT.

**FIG 5:**
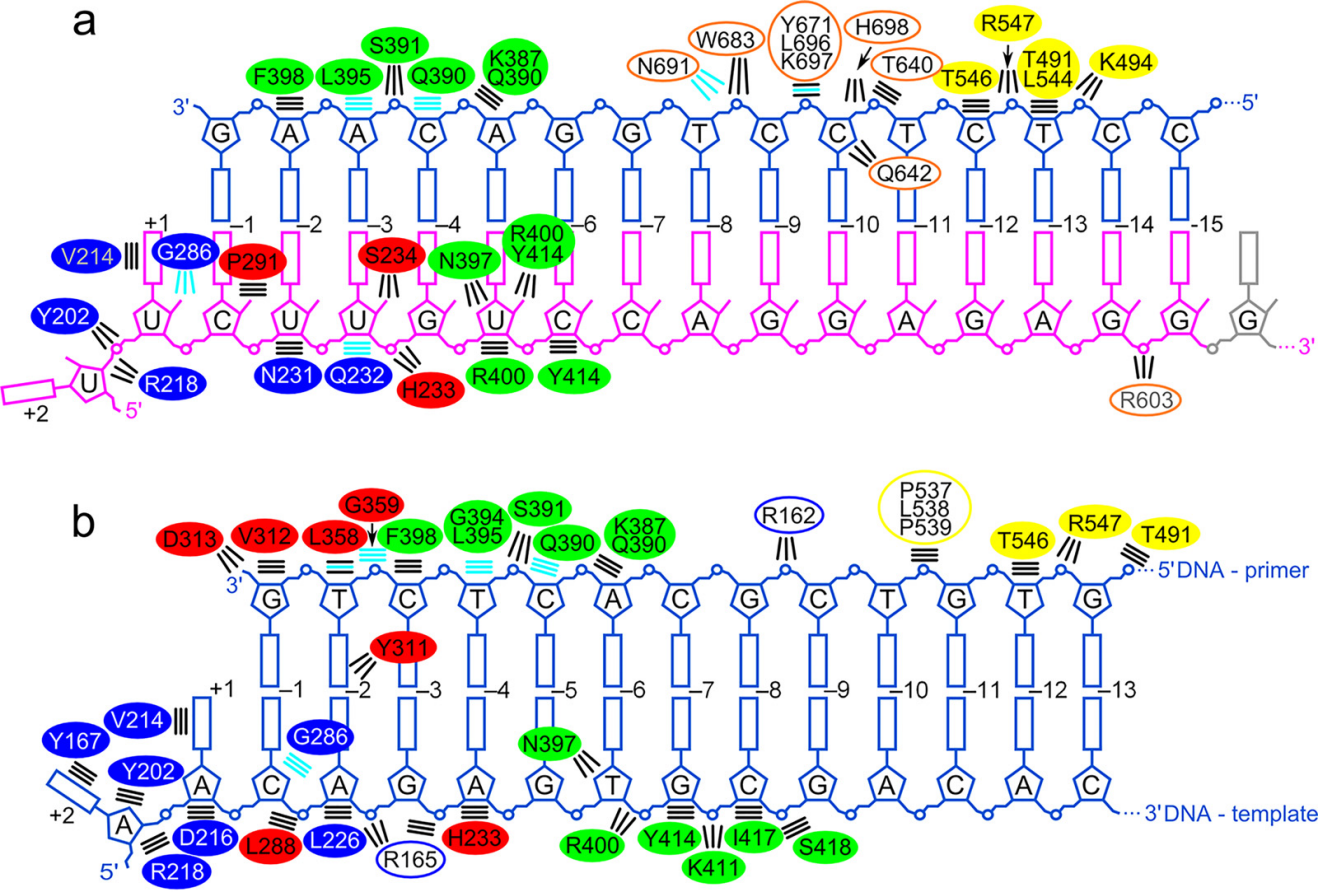
Schematic representation of protein-nucleic acid interactions. (a) Contacts of full-length MFV PR-RT in complex with RNA/DNA. Empty ovals represent interactions that are formed by the RH domain of a symmetry-related protein molecule in the crystal. Oval colors correspond to the coloring of the domains in Fig. 1: blue for fingers, red for palm, green for thumb, yellow for connection, and orange for RH. Radiant lines represent polar contacts. Parallel lines represent van der Waals contacts. Black lines show contacts with side chains. Cyan lines show contacts with the protein backbone. For interactions that are predicted based on the nature of the side chain and distance from the nucleic acid but that are not directly visible in the structures, the residue names/numbers are in gray. (b) Contacts between MFV PR-RT ΔRH and dsDNA. Solid ovals represent residues from subunit A, and empty ovals represent residues from subunit B.

The two crystal structures that are described here provide detailed information about interactions between MFV PR-RT and two types of substrates: RNA/DNA and dsDNA. These interactions are schematically shown in Fig. 5. For the full-length protein, contacts are formed between the POL domain and nt +2 to −6 of the RNA/DNA hybrid (Fig. 5a). The 3′ end of the DNA is located at the POL active site, the architecture of which is very similar to those of other RTs. We note, however, that the 3′ end of the DNA in our structure is slightly displaced from the active site, most likely because of the unusual placement of the base of RNA nt +2 in the grove of the hybrid. The fragment of the substrate that comprises nt −8 to −11 interacts with the RH domain of the symmetry-related molecule. As described below, contacts of the RH domain with this part of the substrate result from crystal packing, and such positioning of this domain cannot be accommodated in a single molecule of MFV PR-RT. Further interactions occur between the connection domain and DNA nt −12 and −13.

In the PR-RT ΔRH structure, protein-dsDNA interactions are observed along almost the entire length of the nucleic acid, and these contacts are mostly mediated by subunit A of the dimer (Fig. 5b). The template strand forms interactions only with the POL domain. The 3′ end of the primer DNA strand is located at the POL active site of subunit A. nt −3 to −6 of the dsDNA are involved in interactions with the thumb subdomain of subunit A. The next substrate region that interacts with the protein involves primer DNA nt −8 and −13, which contact the connection domain of subunits A and B and also Arg162 from the finger subdomain of subunit B.

Several differences are observed in the modes of binding between the RNA/DNA and the dsDNA substrate, which result from different geometries of the double helices of the two types of nucleic acid. In both structures, the thumb subdomain binds the 3′-terminal region of the primer, but the register is shifted, and contacts are rearranged (Fig. 5). In the dsDNA complex, the interactions between the thumb subdomain and the template strand are much more extensive than those in the RNA/DNA complex. Contacts between the 5′-terminal region of the template and the POL domain are similar in both complexes. The same is true for interactions between the 5′-terminal region of the primer and the connection domain (Fig. 5).

### Dimerization upon dsDNA binding also occurs for the full-length enzyme.

**FIG 6:**
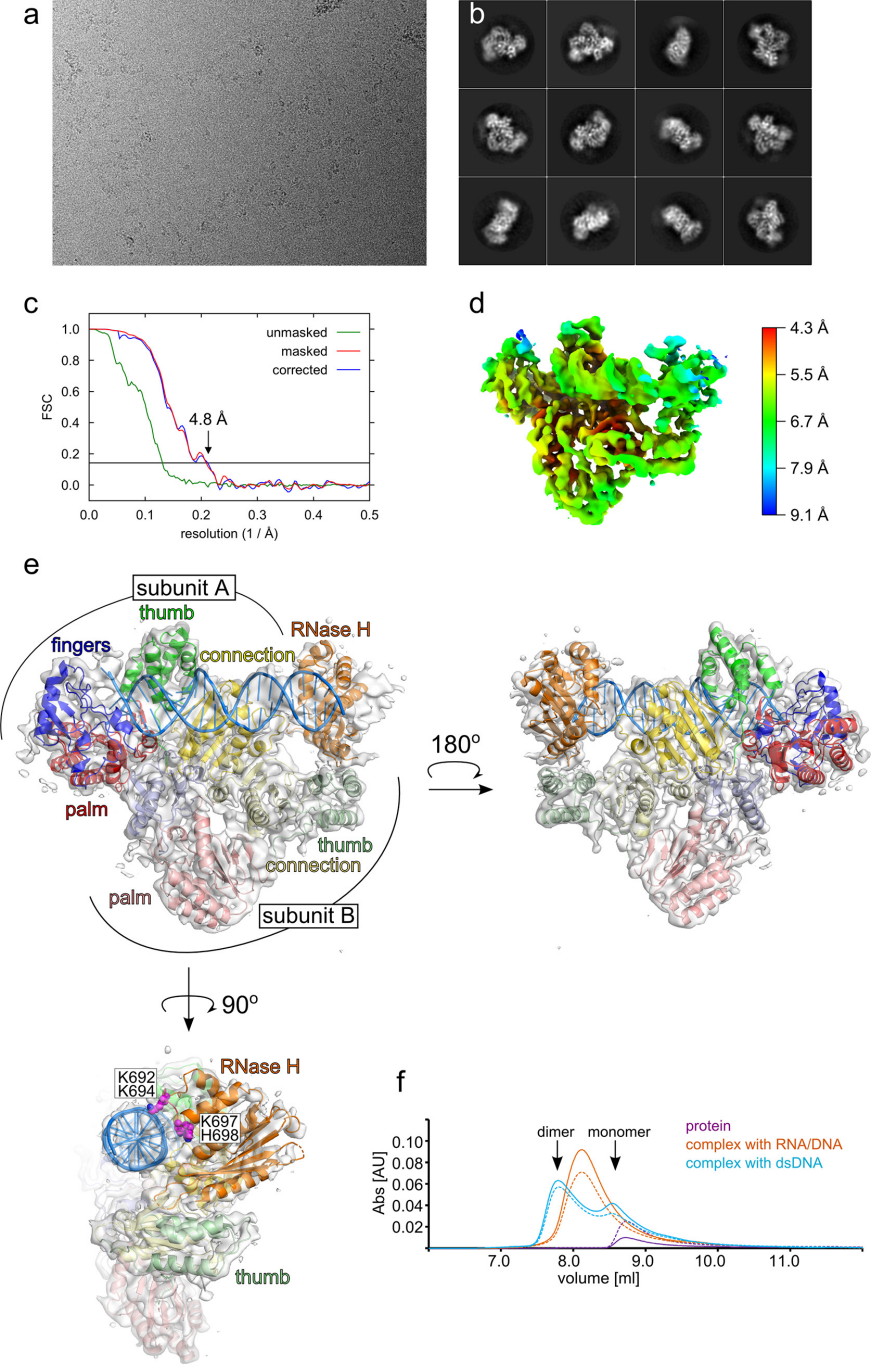
MFV PR-RT dimerization shown by cryo-EM and GF experiments. (a) Example cryo-EM micrograph after motion correction. (b) Typical 2D classes of the MFV PR-RT–dsDNA complex. (c) Gold-standard Fourier shell correlation (FSC) curves between two half-maps generated with RELION postprocessing. The horizontal line represents a value of 0.143. (d) Local resolution calculated from the half-maps in cryoSPARC. (e) Cryo-EM reconstruction of MFV PR-RT in complex with the 22-bp/2-nt-overhang dsDNA substrate (three views). The structure is shown as in Fig. 1, and the cryo-EM reconstruction is shown as a gray surface. Positively charged residues from the basic protrusion are shown as purple spheres. (f) GF/MALS analysis of substrate binding by MFV PR-RT. The trace of the protein alone is in purple. The complex with RNA/DNA is in orange. The complex with dsDNA is in cyan. Solid lines show the absorbance (Abs) at 254 nm. Dashed lines show the absorbance at 280 nm. AU, arbitrary units.

The monomeric form of MFV PR-RT was crystallized using full-length PR-RT in the presence of the RNA/DNA hybrid. The dimeric form was observed in a crystal of truncated PR-RT ΔRH in the presence of a relatively short dsDNA. While PR-RT ΔRH was the only construct that produced crystals of sufficient quality, such a form of the PR-RT is not present in virions. Therefore, it was important to verify whether the protein construct or type of bound nucleic acid determines the multimeric state of the enzyme and to confirm the presence of the dimeric form for the full-length MFV PR-RT present in the virions. To this aim, we used cryo-EM and obtained a 4.8-Å-resolution reconstruction of full-length MFV PR-RT in complex with a longer dsDNA comprising 22 bp and a 2-nt overhang at the 5′ end of the template strand (substrate 3) (see Materials and Methods for the sequence) (Fig. 6a to d). The crystal structure of the PR-RT ΔRH–dsDNA complex was fitted into this cryo-EM reconstruction. Next, the dsDNA substrate was extended, its sequence was changed, and the model was refined using a combination of rigid-body fitting and real-space refinement (Fig. 6e). The reconstruction corresponded well with the dimeric structure of the POL-connection portion of PR-RT ΔRH in complex with dsDNA. The cryo-EM data confirmed that the full-length MFV PR-RT forms dimers upon dsDNA binding. We note that the monomeric form of the MFV PR-RT–dsDNA complex was also likely present in the cryo-EM sample. However, two-dimensional (2D) classes corresponding to this form were not present during data processing, most likely due to the fact that these molecules were too small for particle picking software and proper alignment during classification.

In the cryo-EM reconstruction, we did not observe any density for the protease domains, which indicates that they are more mobile than implied by the crystal structures. However, a density that corresponded to one of the RH domains was observed. Its resolution was lower (Fig. 6d) but sufficient for rigid-body docking of the MFV RH structure. The reconstruction does not clearly show which dimer subunit contributes this RH domain, but the C terminus of the connection domain of subunit A is located closer to the N terminus of this RH domain, so it is a more likely candidate. Interestingly, the RH domain interacts with the thumb subdomain of subunit B, which likely stabilizes the position of the RH domain. Moreover, the RH domain is in the vicinity of the dsDNA substrate, and charged residues Lys692, Lys694, Lys697, and His698 located in the basic protrusion are predicted to form contacts with the nucleic acid (Fig. 6e).

We also prepared cryo-EM samples for MFV PR-RT in complex with a 24-bp RNA/DNA hybrid. However, due to complex aggregation and the small size of the complex, an analysis of these samples was not possible.

To study the oligomeric state of MFV PR-RT further, the protein was mixed with RNA/DNA or dsDNA of the same sequence and length of 34 bp (for sequences, see Materials and Methods) and analyzed by gel filtration coupled to multiangle light scattering (GF/MALS) (Fig. 6f). The full-length protein eluted as a single peak from the GF column with a molecular weight (MW) that was calculated based on MALS of 99.0 kDa (the theoretical MW of the protein is 85 kDa). In the presence of RNA/DNA, we observed a single peak that corresponded to a measured MW of 123.3 kDa (the theoretical MW of the complex of one PR-RT molecule and one RNA/DNA is 105.5 kDa). In the presence of dsDNA, two peaks were observed: a major peak that corresponded to an MW of 171.0 kDa and a second peak that corresponded to an MW of 131.8 kDa (the theoretical MW of a complex that comprises two PR-RT molecules and dsDNA is 190 kDa; the theoretical MW of the complex of one PR-RT molecule with one dsDNA is 105.5 kDa). These results indicated that one protein molecule was present in complex with RNA/DNA, but in the presence of dsDNA, MFV PR-RT exists as a presumably dynamic mixture of two species: monomeric and dimeric.

Our structure may explain why dsDNA but not RNA/DNA promotes the dimerization of the enzyme. The helices of dsDNA and RNA/DNA have different geometries. Consequently, the positioning of the thumb subdomain that tracks the minor grove of the double helix in complex with dsDNA and RNA/DNA differs. The thumb subdomain forms one structural module with the connection domain, so its position is also different for the two types of substrates. The position of the connection domain in the complex with RNA/DNA is incompatible with the binding of subunit B of the dimer, which explains why the protein dimer cannot interact with a hybrid substrate. In other words, the binding of RNA/DNA by subunit A prevents its interaction with subunit B. We also note that in our structures, outside the vicinity of the polymerase active site, we do not observe bending of the nucleic acid, such as has been observed for HIV-1 RT (30). In particular, the 22-bp dsDNA helix in the cryo-EM reconstruction shows only a very small bend of the DNA duplex.

### The mobile RNase H domain performs multiple cuts in the RNA strand.

**FIG 7:**
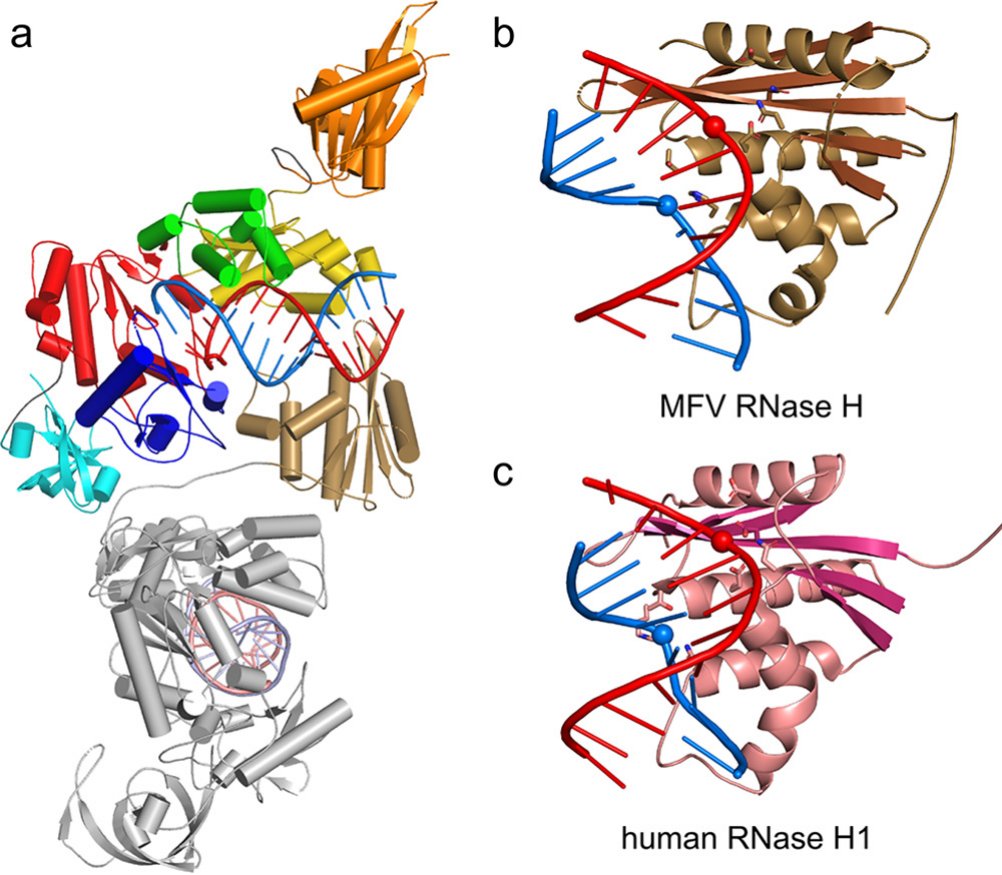
MFV RNase H domain. (a) Structure of full-length MFV PR-RT in complex with RNA/DNA. The main molecule is colored as described in the legend of Fig. 1. The symmetry-related molecule is in gray, with the RH domain in gold and the RNA/DNA substrate in light shades of red and blue. (b) The symmetry-related RH domain of MFV PR-RT that interacts with the RNA/DNA hybrid. The β-strands are in a darker color. Residues that form the active site and phosphate-binding pocket are shown as sticks. The phosphate groups that interact with the active site (RNA) and the phosphate-binding pocket (DNA) are shown as spheres. (c) Human RNase H1 that interacts with the RNA/DNA hybrid (PDB accession no. 2QK9) (27), presented as described above for panel b.

The MFV RH domain is linked to the rest of the structure with a flexible linker. In the full-length structure of MFV PR-RT, it is located further away from the globular part that comprises the PR-POL-connection region and interacting RNA/DNA hybrid (Fig. 1b). However, this hybrid interacts with the RH domain of the symmetry-related MFV PR-RT molecule that is present in the crystal (Fig. 7a). The mode of the interaction between this RH domain and nucleic acid is similar to those of the catalytic complexes of human RNase H1 (Fig. 7b and c). The RNA strand is near a shallow groove on the protein surface, which is located at the edge of the central β-sheet and harbors the active site. In a manner that is very similar to that of the human enzyme, the DNA strand of the hybrid interacts with the two elements that are characteristic of RNases H1, namely, a pocket that tightly binds one of the phosphate groups and a channel between the core of the domain and the basic protrusion. The active site of the symmetry-related RH domain is located in the vicinity of the phosphate of nt −12. This scissile phosphate is, however, displaced from the active site. The distance between the phosphorus atom and C_α_ of Asp598 at the heart of the active site is 10.2 Å versus the expected 7 to 8 Å (27, 31). The observed distance is too long for the proper coordination of catalytic divalent metal ions and for the reaction to proceed.

**FIG 8:**
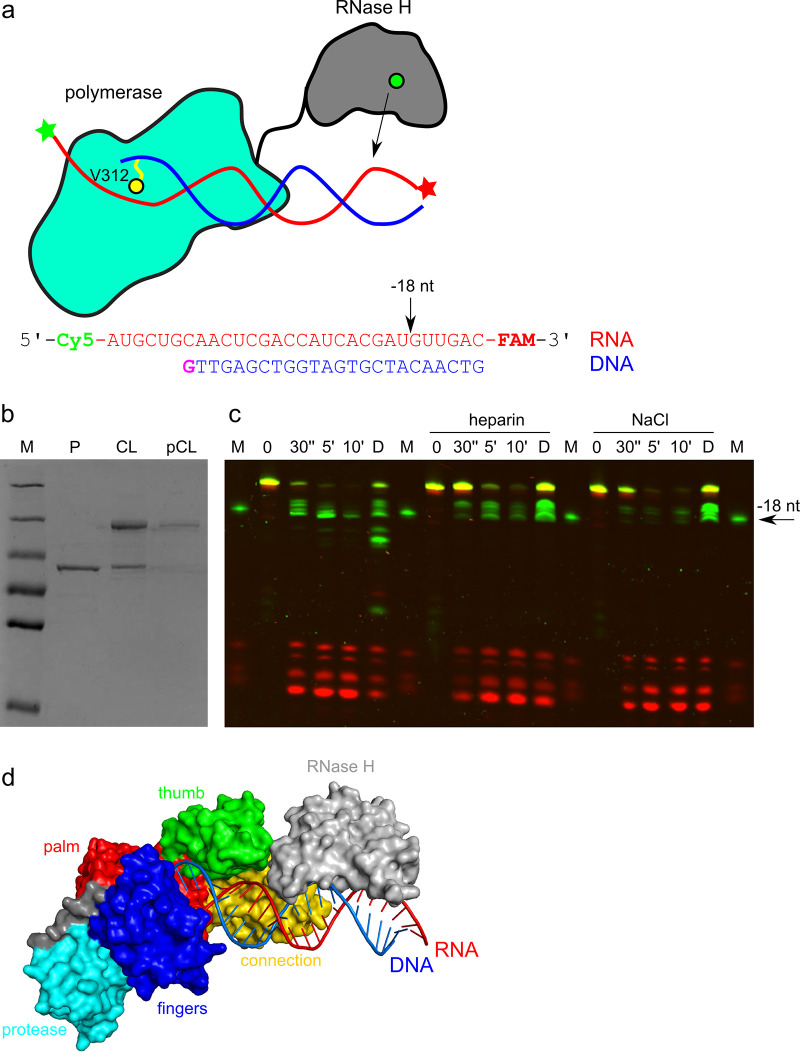
Positioning of the MFV RNase H domain. (a) Schematic of the chemical cross-linking approach. The PR-POL-connection part is shown in green-cyan; the RH domain is in gray, with the active site indicated with a green circle; RNA is in red; and DNA is in blue. V312, which is replaced with cysteine, is shown as a yellow circle, and the disulfide cross-link is shown as a yellow line. The sequence of the RNA/DNA hybrid used for cross-linking is shown below. Guanosine with a thiol-modified base is shown in purple. FAM, 6-carboxyfluorescein. (b) Purification of the chemically cross-linked complex of MFV PR-RT V312C with the RNA/DNA hybrid shown in panel a. P, protein alone; CL, complex after reaction with the modified hybrid; pCL, purified complex. (c) RNase H activity assay using the chemically cross-linked complex. The reaction was started by the addition of Mg^2+^, and aliquots were withdrawn at the times indicated on the top of the gel. Reactions were also performed in the presence of 0.2 mM heparin or 0.5 M NaCl, as indicated on top of the gel. The products were resolved on a TBE-urea gel and visualized by fluorescence. D, reaction in the presence of 20 mM DTT; M, fluorescent RNA marker that corresponds to the products of RH cleavage that would occur 18 nt from the POL active site. This experiment was repeated three times. (d) Model of the MFV PR-RT with the RH domain (gray), whose active site is positioned over the phosphate of nt −18.

We next wished to verify the positioning of the RNase H domain within the full-length MFV PR-RT. For this purpose, we decided to map RNase H cut sites in the RNA/DNA substrate with a recessed 3′ end of the DNA. Such a hybrid would be preferentially bound with the recessed DNA end at the polymerase active site (the configuration observed in our crystal structures). However, binding in other registers cannot be excluded. In order to unequivocally define the RNA/DNA binding register, we used a chemical cross-linking approach (32, 33), which we used previously to study the RNase H activity of HIV-1 RT (34). Briefly, a thiol group is introduced to the guanine base of the DNA, and a protein residue located in its vicinity is replaced with cysteine. Upon protein-nucleic acid complex formation, the thiol on the DNA and the side chain of cysteine are located close enough to react and form a covalent disulfide linkage (Fig. 8a). For our experiments, we chose a modification of nucleotide −1 of the DNA and the V312C amino acid substitution (Fig. 5 and Fig. 8a), similar to the approach that we used previously for HIV-1 RT (34). Upon mixing of the modified hybrid with the V312C variant, an additional slower-migrating band was observed by SDS-polyacrylamide gel electrophoresis (PAGE), which corresponded to a cross-linked complex and which was purified (Fig. 8b) and used for RNase H activity assays. The RNA used in these experiments contained 5′-terminal Cy5 and 3′-terminal fluorescein labels for the visualization of the cleavage products (Fig. 8a). To start the reaction, an Mg^2+^ cofactor was added to the purified cross-linked complex, and the RNase H cleavage products were analyzed by denaturing urea-PAGE (Fig. 8c). We observed that the generated products corresponded to cuts 18, 19, 20, and 21 nt away from the polymerase active site. When dithiothreitol (DTT) was added to the reaction mixture to break the disulfide cross-link, additional products resulting from cleavages closer to the polymerase active site were observed. These cuts were not visible when the formation of noncovalent complexes was inhibited by the addition of heparin or salt at a high concentration. This confirmed that the cuts closer to the polymerase active site result from noncovalent complexes with the RNA/DNA hybrid bound in registers different from the one stabilized by the chemical cross-link.

We performed a modeling exercise in which we positioned the RH domain in various registers on the RNA/DNA hybrid. This modeling revealed that the positioning of the RNase H domain with its active site at nt −18 can be easily accommodated in the full-length enzyme (Fig. 8d). The linker between the connection and RH domains comprises 19 residues that span ∼60 Å in the fully stretched form. Positioning of the RH active site over phosphates of nt −18, −19, −20, and −21 resulted in 28-, 43-, 57-, and 68-Å distances between the C terminus of the connection domain and the N terminus of the RH domain. The first three distances can be spanned by the connection-RH linker, and the positioning over nt −21, which led to a much weaker cut (Fig. 8c), may require a 1-nt translocation of the hybrid within the cross-linked complex. Any placement of the RH domain with its active site interacting with nt 17 to 13 was not possible because of steric clashes with the POL core. The next allowed position was over nt −12, which corresponded to the location of the symmetry-related RNase H domain (Fig. 7a). In this configuration, the distance between the C terminus of the connection domain and the N terminus of the RH domain is ∼60 Å in a straight line, but the protein chain that links the two domains would need to wrap around the connection and RH domains. Thus, the linker may be too short to link the connection and RH domains in the configuration that is observed in the crystal contacts. This is in agreement with the lack of cuts at nt −12 for the cross-linked complex. The fact that this is not an allowed position would also explain why the symmetry-related RH does not form a fully catalytic interaction with the substrate. We conclude that the positioning of the symmetry-related RH domain observed in our crystal cannot be accommodated in a single molecule of MFV PR-RT.

In summary, the structure that is described here allowed the visualization of full-length PR-RT with mobile RH, and the biochemical experiments showed that the RH, which is connected through a flexible linker with the rest of the protein, can cut RNA 18 to 21 nt from the POL active site.

### Conclusion.

In this work, we describe the first structures of foamy viral RT in complex with nucleic acid substrates along with biochemical and biophysical characterization of the enzyme. Spumaretroviruses are a peculiar group that is much more ancient and distantly related to other retroviruses. The unique feature of their RTs is the presence of the N-terminal PR domain. Complete knowledge about the architecture of the full-length foamy viral RT, in particular in complex with nucleic acid substrates, was missing. RTs were previously shown to form monomers (XMRV) (12), constitutive dimers (HIV and Rous sarcoma virus [RSV]) (9, 35), or substrate-induced dimers (Ty3) (14). Our structures showed an unanticipated feature of MFV PR-RT, namely, its ability to adopt both monomeric and dimeric configurations, depending on the substrate to which it binds. The structural and biophysical experiments showed that MFV PR-RT is a monomer upon binding to RNA/DNA and a dimer when it interacts with dsDNA. The physiological importance of this substrate-dependent monomer-dimer switch needs to be further explored using *in vivo* experiments.

## MATERIALS AND METHODS

### Bacterial strains.

The following Escherichia coli strains were used in this study: BL21-Gold(DE3) (Agilent) [E. coli B F^−^ *ompT hsdS*(r_B_^−^ m_B_^–^) *dcm*^+^ Tet^r^ *gal* λ(DE3) *endA* Hte] and Top10 (Thermo Fisher) [E. coli F^−^ *mcrA* Δ(*mrr-hsdRMS-mcrBC*) φ80*lacZ*ΔM15 Δ*lacX74 recA1 araD139* Δ(*ara-leu*)*7697 galU galK rpsL* (Str^r^) *endA1 nupG*].

### Oligonucleotides, plasmids, and mutagenesis.

Synthetic genes of full-length PR-RTs for 4 genera of the *Spumaretrovirinae* subfamily, *Simiispumavirus*, *Bovispumavirus*, *Felispumavirus*, and *Equispumavirus*, were obtained from Bio Basic. Genes of interest were inserted into the pET28-6×His-SUMO expression vector within BamHI and XhoI restriction sites. The QuikChange method (Stratagene) was used to prepare DNA expression constructs that were used for the production of all amino acid point substitution variants of MFV PR-RT: D598N/D668N, N240A/K303A, and V312C. The MFV PR-RT ΔRH expression construct was prepared by PCR. All restriction enzymes were ordered from Thermo Fisher Scientific. DNA KOD polymerase was purchased from Novagen. The DNA purification kit was ordered from Promega.

### Protein preparation.

The expressed proteins contained an N-terminal 6×His tag and the SUMO protein. Protein variants were expressed in E. coli BL21-Gold(DE3) cells overnight at 16°C after induction with 0.4 mM isopropyl β-d-1-thiogalactopyranoside. Bacterial cells were resuspended in buffer containing 40 mM NaH_2_PO_4_ (pH 7.0), 5% (vol/vol) glycerol, 10 mM β-mercaptoethanol, and 20 mM imidazole (buffer 1) with the addition of 150 mM NaCl. Lysozyme at a concentration of 1 mg/ml and a mixture of protease inhibitors were also added. After 30 min of incubation on ice, the concentration of NaCl was increased to 0.5 M, and the suspension was sonicated. To clarify the lysate, it was spun down at 40,000 rpm (45Ti rotor; Beckmann Coulter) and then applied on a His-Trap column (GE Healthcare) that was equilibrated with buffer 1 with 0.5 M NaCl. The column was washed with buffer 1, followed by a wash with the same buffer that included 60 mM imidazole. Proteins were eluted with buffer 1 that contained 500 mM imidazole. To remove the 6×His-SUMO tag, the fusion proteins were digested with a SUMO protease and dialyzed overnight against buffer 1 at 4°C. The cleaved-off SUMO protein was removed on a His-Trap column (GE Healthcare). The flowthrough that contained the tagless proteins was applied to a size exclusion column (Hiload 16/600 Superdex S200; GE Healthcare) that was equilibrated with storage buffer (20 mM HEPES [pH 7.0], 100 mM NaCl, 5% [vol/vol] glycerol, and 1 mM dithiothreitol [DTT]). Pooled fractions were concentrated using an Amicon centrifugal filter device (Millipore), and Tris(2-carboxyethyl)phosphine (TCEP) was added to a final concentration of 2 mM for long-term storage.

The purification procedure for PR-RT ΔRH was similar to the one for the full-length protein but modified by omission of the dialysis step. Before applying the sample onto the second His-Trap column, imidazole was removed by ammonium sulfate precipitation [1 ml of a cold protein solution was precipitated with 0.4 g of (NH_4_)_2_SO_4_]. The same purification procedures were used to purify the SeMet-substituted protein.

### Crystallography.

Crystallization trials for MFV PR-RT were performed using the sitting-drop vapor diffusion method at 18°C. Crystallization trials were prepared for protein alone as well as in the presence of RNA/DNA hybrids and dsDNA ranging from 12 to 26 bp. Before crystallization, protein was mixed with the RNA/DNA hybrid (substrate 1), formed by RNA (5′-UUCUUGUCC**AGGAGAGGG** [the PPT sequence is in boldface type]) and DNA (5′-CCTCTCCTGGACAAG) strands, at a 1:1 molar ratio and a final protein concentration of 5 mg/ml. The best-diffracting crystals were obtained in a Morpheus screen (Molecular Dimensions) with a mixture that contained 10% (wt/vol) polyethylene glycol 8000 (PEG 8000), 20% (vol/vol) ethylene glycol, 0.02 M each alcohol [1,6-hexanediol, 1-butanol, (*RS*)-1,2-propanediol, 2-propanol, 1,4-butanediol, and 1,3-propanediol], and 0.1 M morpholineethanesulfonic acid (MES)-imidazole (pH 6.5).

The crystals of SeMet-substituted protein in complex with substrate 1 were obtained in a Morpheus screen (Molecular Dimensions) with a mixture that contained 10% (wt/vol) PEG 20000, 20% (vol/vol) PEG MME550, 0.02 M each carboxylic acid (sodium formate, ammonium acetate, trisodium citrate, sodium potassium l-tartrate, and sodium oxamate), and 0.1 M MES-imidazole (pH 6.5). These crystals grew in a drop with a 3:1 protein/well solution ratio. The structure of MFV PR-RT was solved by the SAD method using CRANK2 (36) in the CCP4i package (37). The structure was refined in phenix.refine (38) with manual building in Coot (39).

The best-diffracting crystals of MFV PR-RT ΔRH were obtained by the hanging-drop vapor diffusion method for protein at a 5-mg/ml concentration in complex with dsDNA (substrate 2), composed of a template strand (5′-AACAGAGTGCGACAC) and a primer strand (5′-GTGTCGCACTCTG), that was added in a 1:1 molar ratio. The optimal crystallization mixture contained 0.1 M dl-malic acid (pH 7.0) and 10% (wt/vol) PEG 3350 and was initially identified in the PEG/Ion screen (Hampton Research).

All of the X-ray diffraction data sets were collected at the PETRA III storage ring at the P13 beamline operated by EMBL Hamburg (40) and the P11 DESY beamline (41). Data were processed and scaled by XDS (42) or XDSAPP GUI (43). The structure was solved by molecular replacement using the Phaser module (44) in Phenix (38). The truncated structure of MFV PR-RT without the RH domain was used as a search model. The asymmetric unit contained two protein molecules that formed a dimer interacting with the dsDNA molecule. The structure was refined in phenix.refine (38) with manual building in Coot (39). In subunit B, the electron density for the protease domain and the thumb subdomain was less well defined, most likely due to protein mobility. Structural figures were generated using PyMOL (PyMOL molecular graphics system, version 2.2; Schrödinger, LLC [http://pymol.org/]).

### Cryo-electron microscopy.

Full-length MFV PR-RT was mixed with dsDNA (substrate 3) consisting of the template strand (5′-AACAGAGTGCGACACCTGATTCCA) and the primer strand (5′-TGGAATCAGGTGTCGCACTCTG) with 22 bp and a 2-nt overhang at the 5′ end of the template. The reconstituted complex was purified in a glycerol gradient from 5 to 25% (vol/vol). Fractions from the glycerol gradient containing the reconstituted complex were pooled; the buffer was changed to buffer containing 150 mM NaCl, 20 mM HEPES (pH 7.0), and 1 mM DTT; and the sample was concentrated on an Amicon centrifugal filter device (Millipore) to approximately 0.6 mg/ml. The concentrated sample (3 μl) was applied to a glow-discharged grid (Quantifoil R2/1 on 200 gold mesh, X-102-Au200; Jena Bioscience) and vitrified in liquid ethane using an FEI Vitrobot Mark IV instrument (Thermo Fisher Scientific) with the following settings: temperature of 4°C, 95% humidity, blot time of 4 s, and blot force of 0.

Data collection was performed on a Titan Krios G3i electron microscope (Thermo Fisher Scientific) operating at 300 kV and equipped with a BioQuantum energy filter (with a 20-eV energy slit) and a K3 camera (Gatan) at the Solaris National Synchrotron Radiation Centre in Krakow, Poland. Movies were collected with EPU software using aberration-free image shift (AFIS) at a nominal magnification of ×105,000 corresponding to a physical pixel size of 0.86 Å. The nominal defocus range was set to −0.8 to −2.0 μm, the total dose (fractionated into 40 frames) was 50 e/Å^2^, and the dose rate was 16 e per pixel per s. To overcome the orientational bias, the stage was tilted to 30° during data collection.

### Cryo-electron microscopy data processing.

Collected micrographs were processed with RELION-3.1 (45) and cryoSPARC (46). A total of 3,792 raw movies were motion corrected and binned 2× using the RELION implementation of MotionCor2 software (47). The contrast transfer function (CTF) was fitted with CTFIND-4.1 (48). The initial set of 240,847 particles, picked with crYOLO (49), was used to create 2D templates for particle picking via 2D classification in RELION and the initial three-dimensional (3D) model in cryoSPARC. A total of 344,421 particles were picked with the template-based autopicking algorithm in RELION, binned 2× to a pixel size of 3.44 Å/pixel, and subjected to two rounds of reference-free 2D classification. A total of 162,211 selected particles were reextracted with 1.72 Å/pixel and subjected to two rounds of 3D classification (heterogeneous refinement) in cryoSPARC. A total of 69,722 selected particles were reimported into the RELION pipeline with scripts from UCSF pyem (50) (UCSF pyem v0.5; Zenodo) for 3D refinement, the first round of Bayesian polishing (with reextraction with an unbinned pixel size of 0.86 Å/pixel), and per-particle CTF refinement. After another 3 rounds of 3D classification in cryoSPARC (one unsupervised/*ab initio* reconstruction and two heterogeneous refinements), 45,454 selected particles were once again reimported into RELION and subjected to two additional rounds of Bayesian polishing followed by CTF refinement (this time with additional correction of beam tilt and anisotropic magnification per exposure group), which yielded a map at a 4.9-Å resolution. To improve the quality of the resulting map, an additional 3 rounds of 3D classification were performed in RELION, followed by a final round of Bayesian polishing and CTF refinements and an additional round of 2D classification. The final 3D refinement of 20,071 selected particles produced a map at a 4.8-Å resolution, with visually better quality than the previous 4.9-Å map. The resolution was estimated from gold-standard-masked Fourier shell correlation (FSC) at the 0.143 threshold, and the local resolution was calculated from the half-maps in cryoSPARC. The final map was sharpened in RELION with a *B* factor of 134 Å^2^. The data collection and refinement statistics are shown in Table 2.

**TABLE 2:** Cryo-EM data collection and processing

Parameter	Value
Microscope	Titan Krios G3i
Camera	Gatan K3
Energy filter	Gatan BioQuantum with 20-eV slit
Voltage (kV)	300
Magnification	×105,000
Electron exposure (e/Å^2^)	50
Defocus range (μm)	−0.8 to −2.0
Tilt angle (°)	30
Pixel size (Å)	0.86
Symmetry imposed	C_1_
No. of initial particle images	344,421
No. of final particle images	20,071
Resolution at 0.143 FSC (masked) (Å)	4.8
Map-sharpening *B* factor (Å^2^)	134

The crystal structure of MFV PR-RT ΔRH in complex with dsDNA was fitted into the cryo-electron microscopy (cryo-EM) reconstruction. The part of the model corresponding to the PR domain not visible in the reconstruction was removed, dsDNA was extended, and its sequence was changed to the one corresponding to the used nucleic acid substrate. The model was next refined using rigid-body fitting of its segments and real-space refinement in Coot (39) and Phenix (38).

### RNase H activity within the cross-linked MFV PR-RT–hybrid complex.

The DNA oligonucleotide (5′-GTCAACATCGTGATGGTCGAGTT**G**) for chemical cross-linking was obtained from Metabion (Martinsried, Germany). The base at the desired position (in boldface type) was changed to 2-F-dI. Thiol-modified DNA was prepared by the replacement of the fluorine atom in the base with an amine group by the addition of cystamine as previously described (34). A fluorescently labeled cRNA oligonucleotide (5′-AUGCUGCAACUCGACCAUCACGAUGUUGAC) was ordered from Future Synthesis (Poznan, Poland) and dissolved in water with 0.5 mM EDTA. RNA and DNA oligonucleotides were annealed in a solution containing 10 mM Tris (pH 7.8) and 25 mM KCl by heating to 90°C for 3 min, followed by cooling to 4°C.

A chemical cross-linking reaction was performed by mixing 3.5 μM protein and 8.75 μM substrate in a buffer with 50 mM Tris (pH 7.4), 25 mM NaCl, 25 mM KCl, 30% (vol/vol) glycerol, and 1 mM DTT and incubating the mixture at 37°C for 2 h, followed by 14 h at 24°C. The cross-linking reaction mixture was applied to a Superdex 200 Increase 10/300 GL column (GE Healthcare) equilibrated with a buffer containing 100 mM NaCl, 20 mM HEPES (pH 7.0), and 5% (vol/vol) glycerol. The complex was next loaded onto a heparin column equilibrated with the same buffer. Elution of a cross-linked complex was performed by increasing the concentration of NaCl from 100 mM to 1 M.

For the analysis of RNase H cleavage, samples contained 60 nM the MFV PR-RT–RNA/DNA-cross-linked complex in a solution containing 20 mM Tris (pH 8.0), 100 mM NaCl, and 1 mM DTT. The reactions were initiated by the addition of 8 mM MgCl_2_ at 37°C, and the reaction was stopped at selected time points by the addition of an equal volume of 7 M urea–1× Tris-borate-EDTA (TBE) and heating to 95°C for 2 min. To inhibit the formation of noncovalent complexes, reactions were also conducted in the presence of 0.5 M NaCl or 0.2 mM heparin. For control reactions, 20 mM DTT was added to the sample. The reaction products were analyzed by denaturing polyacrylamide gel electrophoresis (PAGE) (20% TBE-urea gel). The products were visualized on an Amersham Typhoon RGB biomolecular imager with ImageQuant Total Lab software (GE Healthcare).

### Gel filtration/multiangle light scattering.

For substrate-binding assays, the RNA/DNA substrate (5′-GUGAGCGAACAGAGUGCGACACCUGAUUCCAUGA [RNA]/5′-TCATGGAATCAGGTGTCGCACTCTGTTC [DNA]) and **a** dsDNA substrate (5′-GTGAGCGAACAGAGTGCGACACCTGATTCCATG/5′-TCATGGAATCAGGTGTCGACTCTGTTC) were used. MFV PR-RT was mixed with nucleic acid at a 2:1 molar ratio and applied to an XBridge protein bridged ethylene hybrid size exclusion chromatography (BEH SEC) column (Waters) that was equilibrated with a solution containing 20 mM HEPES (pH 7.0), 250 mM NaCl, 5% (vol/vol) glycerol, 0.5 mM EDTA, and 1 mM DTT. Absorption was monitored at 254 nm and 280 nm. The molecular weight of the eluted species was determined using MALS on Optilab T-rEX and Dawn Heleos II systems (Wyatt Technology).

### Data availability.

All data needed to evaluate the conclusions in the paper are present in the paper. Atomic coordinates for MFV PR-RT–substrate complex structures were deposited in the Protein Data Bank under accession no. 7O0G and 7O0H for full-length MFV PR-RT in complex with RNA/DNA and MFV PR-RT ΔRH in complex with dsDNA, respectively. Cryo-EM potential maps have been deposited in the EMDB under accession number EMD-12698 (https://www.emdataresource.org/EMD-12698), and atomic coordinates have been deposited in the PDB under accession no. 7O24.
